# Research on the Performance and Computational Fluid Dynamics Numerical Simulation of Plate Air Gap Membrane Distillation Module

**DOI:** 10.3390/membranes14080162

**Published:** 2024-07-24

**Authors:** Haojie Bi, Hongying Yuan, Zhiyuan Xu, Zhuobin Liang, Yongliang Du

**Affiliations:** Tianjin Key Laboratory of Aquatic Science and Technology, School of Environmental and Municipal Engineering, Tianjin Chengjian University, Tianjin 300384, China; bi15532393234@163.com (H.B.); yuanhy_00@163.com (H.Y.); xlegionx@163.com (Z.X.); liangzhuobin0914@163.com (Z.L.)

**Keywords:** air gap membrane distillation, heat transfer, computational fluid dynamics, temperature polarization, concentration polarization

## Abstract

Membrane distillation (MD) is widely used in the field of seawater desalination. Among its various sub-categories, air gap membrane distillation (AGMD) stands out due to its high thermal efficiency and compatibility with low-grade heat sources. This study delves into the impact of varying operating conditions on AGMD performance, employing numerical simulations which are grounded in experimental validation. The objective was to enhance the performance of AGMD, mitigate polarization phenomena, and provide a reference for optimizing membrane component design. The results show that the agreements between the simulated and the experimental values were high. When increasing the feed temperature and decreasing the coolant temperature, the impact of polarization phenomena on the performance of AGMD was reduced. The mass flux, Total Permeate Concentration (TPC), and heat flux increased by 81.69%, 36.89%, and 118.01%, respectively, when the feed temperature was increased from 50 °C to 75 °C. When the coolant temperature decreased from 22 °C to 7 °C, the mass flux increased by 37.06%. The response surface analysis revealed that the feed temperature has significant influence on AGMD performance, and there is a noticeable interaction between the feed temperature and coolant temperature. These findings will play key roles in practical applications.

## 1. Introduction

Desalination is considered to be an important technology to alleviate or potentially solve the shortage of fresh water resources. Membrane treatment, as the main desalination technology, includes reverse osmosis, electrodialysis, MD, and other technologies.

MD is an emerging separation technology that capitalizes on the vapor pressure disparity between the two sides of a membrane. It has many advantages, e.g., mild operating conditions, less energy consumption, and the utilization of waste heat; thus, it is considered to have the most promising development prospects. Over the past few decades, most studies have been focusing on the application of direct-contact membrane distillation (DCMD) for wastewater treatment, seawater desalination, recycling, and the concentration of volatile substances [1,2,3,4]. Due to its simple structure and large mass flux, DCMD tends to attract more research interest [5,6]. On the other hand, the direct contact between the membrane and liquid during the DCMD process would cause a large amount of heat loss, resulting in low thermal efficiency [7]. In comparison, AGMD can reduce heat loss during operation as well as have higher thermal efficiency, which has gained more attention from scholars.

Different from DCMD, AGMD inserts a layer of stagnant air gap between the membrane and the condensing plate. The water vapor passes through the air gap and condenses on the condensing plate. The presence of the air gap in AGMD mitigates the risk of membrane contamination and minimizes heat loss resulting from heat conduction from the hot side of the feed liquid [8]. Meanwhile, the mass transfer resistance is also indeed increased for AGMD, causing a lower flux. Nevertheless, it is considered that AGMD will the most widely used membrane treatment method in the future. At present, many researchers are focusing on how to improve its mass flux and prevent membrane contamination [9,10,11]. Studies show that there are many factors affecting the MD process; the most important ones are the operating conditions and membrane materials, such as temperature, flow rate, concentration, membrane materials, and porosity. Due to the complexity of the MD process, the experimental method cannot account for all factors. The experiment process is also limited by flow field disturbance, external environment changes, and measurement accuracy, which will affect the experimental results to varying degrees. In addition, MD is a thermally driven process that entails concurrent heat and mass transfer; thus, the influence of temperature and concentration polarization on the system cannot be ignored. If only relying on the experiment, the distribution of the heat boundary layer and concentration boundary layer near the membrane surface cannot be obtained.

Computational fluid dynamics (CFD) is often used to simulate complex fluid flow and heat transfer processes. The numerical simulation method not only avoids experimental errors but also informs the design and optimization of membrane components. Janajreh et al. [12] used CFD to simulate AGMD and DCMD models and compared their performances. The results showed that due to the existence of an air gap, the mass flux of AGMD was lower than DCMD. These simulation results have not been verified by experiments. Tan et al. [13] used CFD to build a 3D model, to simulate the effects of three different types of gaskets (nickel, copper, and foam) on the MD performance. The simulation results indicated that a metal gasket improves the thermal efficiency of the MD process. Ali et al. [14] developed a two-dimensional phase-change porous media mass transfer model based on the molecular diffusion theory to simulate the AGMD mass transfer and heat transfer processes using different porosities and membrane materials. The results showed that the model could accurately simulate the AGMD process. Karbasi et al. [15] utilized numerical simulation to model a novel disk-shaped module with radiation baffles, and the findings showed that the new membrane module could increase the mass flux by approximately 6%. Darman et al. [16] employed CFD technology to investigate the impact of membrane surface morphology on the DCMD process. The results indicated that the ripple-shaped protrusions on the membrane surface can enhance water flux and reduce the temperature polarization coefficient in the distillation process. Cipollina et al. [17] added different types of diaphragms in the simulation process to investigate their effect on momentum and heat transfer in the MD process. By applying a fixed value of heat flux on the membrane surface, the transmembrane heat transfer process was simulated. It turns out diaphragms can alter the flow state of liquid and promote its flow from the material liquid side to the membrane surface to produce a higher mass flux. In the numerical simulation process, certain parameters are idealized, and some influencing factors are not fully considered, such as the micro-structure and surface characteristics of the membrane, the heat efficiency of the MD module, as well as water condensate film over the condenser plate. In future in-depth research, it will be necessary to consider the actual conditions of the experimental process more comprehensively.

Currently, CFD technology has been widely applied in the field of MD and achieved numerous research accomplishments. However, only limited studies exist when it comes to the significance of membrane surface polarization phenomena. Therefore, this study combines experimental data with simulation results to conduct experimental research on the performance of flat-plate AGMD components. A 2D model was developed in CFD to simulate the AGMD process, with the heat and mass transfer processes of the membrane surface controlled by User-Defined Functions (UDFs). There was also an exploration of parameters such as the TPC and Concentration Polarization Coefficient (CPC). Furthermore, the response surface methodology (RSM) was used to analyze the extent to which different operating conditions affect the performance of membrane distillation.

## 2. Theoretical Model

### 2.1. Heat Transfer Mechanism

**Figure 1:**
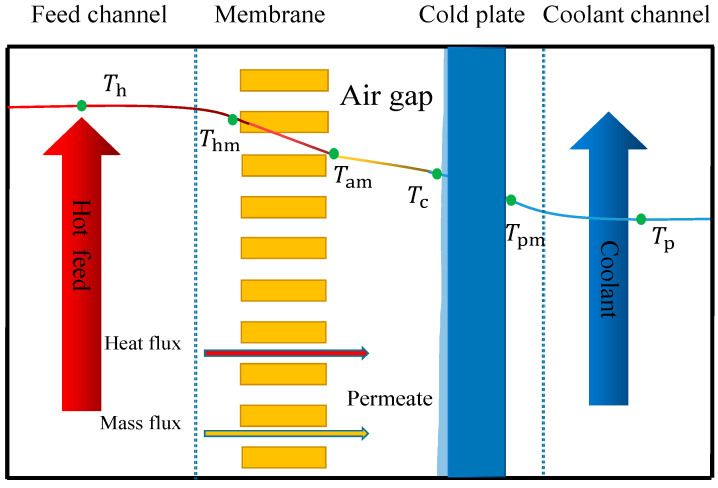
Heat and mass transfer process of AGMD.

Figure 1 reveals the heat and mass transfer process of AGMD. In the AGMD system, the heat first passes through the boundary layer of the liquid on the surface of the membrane, where the volatile components evaporate. The steam passes through the membrane holes and air gaps, and some heat is transferred in the form of conduction. Then, the steam condenses on the surface of the condensing plate to form a water condensate film, and the heat is transferred to the coolant through the condensing plate and the condensate film. Eventually, the heat enters the main phase of the coolant through the boundary layer on the surface of the condensing plate. Due to the complexity of the AGMD system in the actual process, it is challenging to take all factors into account in the simulation; therefore, some reasonable assumptions are made for the model. The assumptions are as follows:

The AGMD process is at steady state.

Effects of gravity are not considered.

Only water vapor can pass through the membrane. Non-volatile components cannot. The retention rate of the membrane is 100%.

The effect of water condensation film on heat transfer is disregarded.

The membrane surface structure and fouling are not considered.

Heat transfer happens regardless of the external environment.

In the process of AGMD, water molecules pass through the boundary layer of the membrane surface, from the main body of the liquid to the membrane surface, where vaporization occurs. Due to the hydrophobicity of the membrane, the membrane pores on the surface are not wetted. Water enters the membrane pores in the form of vapor and transfers inside the pore. The heat transfer *Q_h_* in the feed boundary layer can be calculated by Equation (1).

(1) $$Q_{h}=h_{h}(T_{h}-T_{hm})$$

where *h_h_* is the convective heat transfer coefficient on the feed liquid side; *T_h_* is the main body temperature of the feed liquid; *T_hm_* is the membrane surface temperature on the feed liquid side.

The total transmembrane heat (*Q_m_*) includes sensible heat (*Q_m_*_,*s*_) and latent heat (*Q_m_*_,*l*_).

(2) $$Q_{m}=Q_{m,s}+Q_{m,l}$$

*Q_m_*_,*s*_ is the heat conducted by the membrane itself and the transferred heat inside the water vapor molecule.

(3) $$Q_{m,s}=\frac{k_{m}}{b_{m}}(T_{hm}-T_{am})$$

where *k_m_* is the membrane heat conduction coefficient; *b_m_* is the membrane thickness; *T_hm_* is the feed liquid-side membrane surface temperature; *T_am_* is the air gap-side membrane surface temperature.

*Q_m_*_,*l*_ is the heat absorbed by a water molecule on the feed liquid-side surface, as it changes from liquid to gas.

(4) $$Q_{m,l}=N\cdot \Delta H_{1}$$

where *N* is the mass flux; Δ*H* is the latent heat of water evaporation at the surface of membrane temperatures.

The total heat transfer in the air gap (*Q_g_*) includes sensible heat (*Q_g_*_,*s*_) and latent heat (*Q_g_*_,*l*_). *Q_g_*_,*s*_ is composed of air heat conduction. *Q_g_*_,*l*_ is composed of the steam molecular diffusion.

(5) $$Q_{g}=Q_{g,s}+Q_{g,l}=\frac{k_{l}}{b_{l}}(T_{am}-T_{c})+N\cdot \Delta H_{2}$$

where *k_l_* is the heat conduction coefficient of water vapor and air mixture; *b_l_* is air gap thickness; *T_am_* is the air gap-side membrane surface temperature; *T_c_* is the temperature of the condensation surface.

*Q_a_* is the heat going through the condensing plate, which is calculated as follows:

(6) $$Q_{a}=\frac{k_{a}}{b_{a}}(T_{c}-T_{pm})$$

where *k_m_* is the condenser plate conduction coefficient; *b_a_* is the condensing plate thickness; *T_pm_* is the temperature of the condensing wall on the coolant side.

The heat transferred by the membrane surface on the coolant side is the same as that on the feed liquid side, and the calculation formula is as follows:

(7) $$Q_{p}=h_{p}\left(T_{p}-T_{pm}\right)$$

where *h_p_* is the convection heat transfer coefficient on the coolant side. *T_p_* is the body temperature of the coolant side.

### 2.2. Mass Transfer Mechanism

Mass flux typically refers to the multiplication of the mass transfer coefficient and the saturated vapor pressure difference across the membrane [18]. The mass transfer mechanism can be explained by molecular diffusion, Knudsen diffusion, and viscous flow diffusion. However, a composite mass transfer model that integrates these three different diffusion types is typically employed for a more accurate depiction [19,20]. Due to the low solubility of air in water, it is assumed that air is a stagnant membrane; thus, the effect of viscous flow is ignored [21]. The formula to calculate the mass flux is as follows:

(8) $$N=k(P_{fm}-P_{am})$$

(9) $$P_{fm}=(1-x_{s})P_{hm}\gamma_{W}$$

(10) $$\gamma_{W}=1-0.5x_{s}-10x_{s}^{2}$$

(11) $$P_{hm}=\exp(23.1964-\frac{3816.44}{T_{hm}-46.13})$$

(12) $$P_{am}=\exp(23.1964-\frac{3816.44}{T_{am}-46.13})$$

where *N* is the mass flux; *k* is the total mass transfer coefficient; *P_hm_* is the saturated vapor pressure of water on the feed liquid side; *P_fm_* is the vapor pressure of water; *x_s_* is the molar fraction of NaCl in the solution; *γ_w_* is the activity coefficient of water; *P_am_* is the saturated vapor pressure of water on the air gap side. When the NaCl concentration is relatively low, the activity coefficient approaches 1 and *P_fm_* is essentially equal to *P_hm_*. The saturated vapor pressure (*P_hm_*) was used as the vapor pressure on the feed side during the simulations in this study.

The molecular diffusion coefficient is as follows:

(13) $$K_{m}=\frac{D_{ij}p\varepsilon M_{v}}{\tau \delta RT_{M}}$$

where *D_ij_* and *τ* can be calculated according to the following empirical formula [22]:

(14) $$D_{ij}p=1.895\times 10^{-5}T^{2.072}$$

(15) $$\tau =\frac{{\left(2-\varepsilon \right)}^{2}}{\varepsilon }$$

where *D_ij_* is the diffusion rate of water molecules in the air. *p* is pressure; *ε* is porosity; *M_v_* is gas molecular weight; *τ* is Tau zigzag factor; *δ* is membrane thickness; *R* is gas constant; *T_M_* is the average temperature on both sides of the membrane.

The Knudsen diffusion coefficient is as follows:

(16) $$K_{k}=\frac{2}{3}\frac{r\varepsilon }{\tau \delta }{\left(\frac{8M_{v}}{\pi RT_{M}}\right)}^{0.5}$$

The total mass transfer coefficient is as follows:

(17) $$k=\frac{K_{k}\cdot K_{m}}{K_{k}+K_{m}}$$

### 2.3. Polarization Coefficient

When the MD system is actually operating, the temperature of the membrane surface is lower than that of the main body of the feed liquid due to the velocity gradient at the membrane surface. Consequently, the vapor pressure difference across the membrane is reduced, leading to a decrease in mass flux. This phenomenon is usually referred to as temperature polarization, which can be measured by the *TPC.* Due to the material exchange on both sides of the membrane, water vapor molecules enter the air gap through the membrane pores, leading to an increased concentration at the membrane surface compared to the main part and an increase in mass transfer resistance. This is the concentration polarization, which can be measured by *CPC*. Such polarization will decrease the mass flux [23,24] or even lead to the blockage of membrane pores, which will affect the efficiency of water production.

(18) $$TPC=\frac{T_{hm}-T_{am}}{T_{h}-T_{p}}$$

(19) $$CPC=\frac{w_{hm}}{w_{h}}-1$$

where *w_hm_* is the membrane surface mass fraction; *w_h_* is the main mass fraction of the feed liquid.

## 3. Experimental Process

**Figure 2:**
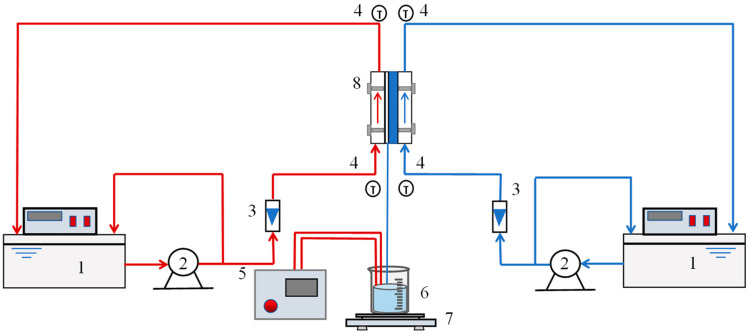
Flow chart of AGMD experimental device. 1. Thermostatic water tank. 2. Circulating pump. 3. Flowmeter. 4. Thermometer. 5. Conductivity meter. 6. Water production. 7. Balance. 8. AGMD membrane module.

The flow diagram of the AGMD experimental device is shown in Figure 2. A thermostatic water tank (1, DF-101T-5, Shenzhen Dingxinyi Laboratory Equipment Co., Ltd., Shenzhen, China) was used to maintain the feed liquid at a controlled temperature. A pump (2, MP-20RM, Shanghai Maike Pump Industry Manufacturing Co., Ltd., Shanghai, China) was used to circulate the feed liquid on the shell side of the membrane. The liquid flow rate was monitored by a liquid flowmeter (3, LZB-3WB, Xiangjin Flow Meter Factory, Taizhou, China), before being channeled into the AGMD module. The dimensions of the hot-side and cold-side flow channel within the membrane module were as follows: a length of 320 mm, a width of 8 mm, and a height of 8 mm. The effective membrane area within the module was 2560 mm^2^, and the air gap thickness within the module was 3 mm. The coolant circulation followed the same principle as the feed liquid side. The temperature of the feed liquid and coolant could be controlled by the thermostatic water tank to remain constant within a range of 0–100 °C. In the AGMD process, the transferred water vapor was condensed on the condensing plate surface. Once the system reached a steady state, the mass of the produced water was determined at ten-minute intervals using an electronic balance (7, JY5002N, Mettler-Toledo Instruments Co., Ltd., Shanghai, China), and the average of five measurements was used to calculate the membrane flux. The conductivity of the produced water was measured using a conductivity meter (5, DDSJ-319, Leici Sensor Technology Company, Shanghai, China). The inlet and outlet temperatures of the both the feed and cold solution were monitored by four temperature detectors (4, EX6000, YiLi Technology, Guangzhou, China). The membrane material used in the experiment was PTFE-PVDF (Shanghai Minglie Membrane Company, Shanghai, China).

## 4. Numerical Simulation

### 4.1. CFD Model Governing Equations

**Figure 3:**
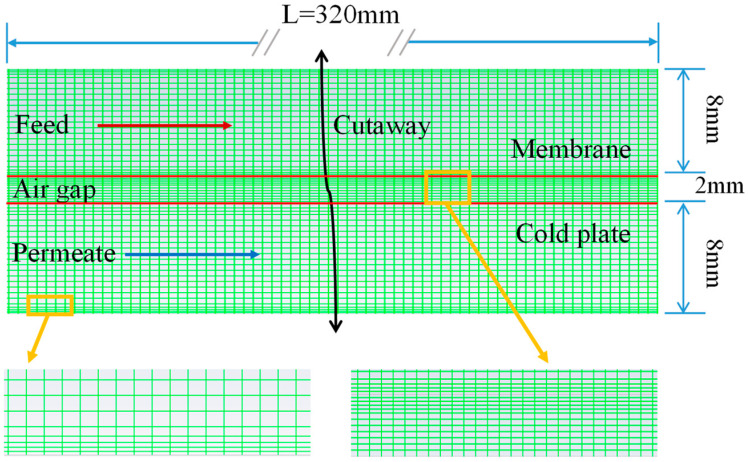
AGMD numerical model setup and discretized mesh.

A 2D model is built based on the actual membrane components. The fluid dynamics software FLUENT 2021 is used to simulate the AGMD process with high salt concentration. The physical property parameters of the experimental membrane are presented in Table 1. The AGMD model is composed of three calculation domains. The hot and cold runner is 320 mm in length and 8 mm in height, and the air gap is 320 mm in length and 2 mm in height (see Figure 3). A total of 185,107 grid units are divided. The membrane is located between the feed liquid channel and the air gap and the aluminum plate between the air gap and the coolant channel. The membrane and the aluminum plate are treated without thickness values (membrane thickness, mass transfer, and heat transfer processes is defined in UDFs and aluminum plate thickness in Fluent software).

For stable and incompressible Newtonian fluids, this numerical system is governed by the conjugated steady incompressible Navier–Stokes equations, energy equations, and continuity equations. These equations are written as follows:

Continuity [25]:

(20) $$\frac{\partial \rho V_{x}}{\partial_{x}}+\frac{\partial \rho V_{y}}{\partial_{y}}=S_{m}$$

*x*-Momentum [25]:

(21) $$V_{x}\frac{\partial \rho V_{x}}{\partial_{x}}+V_{y}\frac{\partial \rho V_{y}}{\partial_{y}}=-\frac{\partial \rho }{\partial x}+\mu (\frac{\partial^{2}V_{x}}{\partial x^{2}}+\frac{\partial^{2}V_{x}}{\partial y^{2}})$$

*y*-Momentum [25]:

(22) $$V_{x}\frac{\partial \rho V_{y}}{\partial_{x}}+V_{y}\frac{\partial \rho V_{y}}{\partial_{y}}=-\frac{\partial \rho }{\partial y}+\mu (\frac{\partial^{2}V_{y}}{\partial x^{2}}+\frac{\partial^{2}V_{y}}{\partial y^{2}})$$

Energy [25]:

(23) $$V_{x}\frac{\partial \rho C_{P}T}{\partial_{x}}+V_{y}\frac{\partial \rho C_{P}T}{\partial_{y}}=k\left(\frac{\partial^{2}T}{\partial x^{2}}+\frac{\partial^{2}T}{\partial y^{2}}\right)$$

where *V_x_* and *V_y_* are the velocity components, *ρ* is the density, *μ* is the viscosity, *C_p_* is the specific heat, *k* is the thermal conduction coefficient, and *S_m_* is the lost mass of water vapor.

### 4.2. UDFs

In this study, the mass and heat transfer process of AGMD is established by FLUENT’s UDF. Predefined macros provided by FLUENT Inc. and the DEFINE_ADJUST macro are used to mark the mesh on both sides of the membrane surface. DEFINE_SOURCE is used to control the heat transfer and mass transfer process on the membrane surface. Through these predefined macros, data can be obtained via the FLUENT solver. Please refer to the Supplementary Materials for the UDF code.

### 4.3. Boundary Conditions

The boundary conditions define the velocity inlet and pressure outlet for both the feed liquid and coolant sides. They also specify the inlet velocity, temperature, and outlet pressure, with the pressure size being normal. There is no penetrating velocity on the top and lower surfaces of each membrane component. A UDF is introduced into the first layer mesh of the membrane, where both the mass source terms are applied to realize the trans-membrane heat transfer and mass transfer process. The inlet velocity of feed and coolant is set at 0.068 m/s. The temperature of the feed liquid side is set at 50 °C, 55 °C, 60 °C, 65 °C, and 70 °C, and the coolant side is set at 7 °C. In the simulation process, FLUENT’s separated pressure base implicit solver is used, and the time format is fixed normally. The second-order upwind scheme is used to discretize variables such as pressure and momentum, and the coupling of pressure and velocity is solved by a simple algorithm.

Table 2 shows all the boundary conditions listed. Table 3 indicates the salt water/feed, air, cold plate, coolant, and membrane properties, which are sourced from the Fluent software.

## 5. Results and Discussion

### 5.1. Cell Independence Test

The principle of numerical simulation is to obtain accurate calculation results with fewer cells. In this study, calculation results of cells 43,016, 93,049, 185,107, and 987,315 are cross-compared. Table 4 shows that the mass flux and feed liquid outlet temperature of cells 185,107 and 987,315 are essentially unchanged, with differences of only 0.38% and 0.03% respectively. Therefore, the cell number no longer affects the calculation result. Based on this, the subsequent simulation adopts the calculation results of cell 185,107.

### 5.2. Verification of the CFD Model

#### 5.2.1. Mass Flux at Different Feed Temperatures

**Figure 4:**
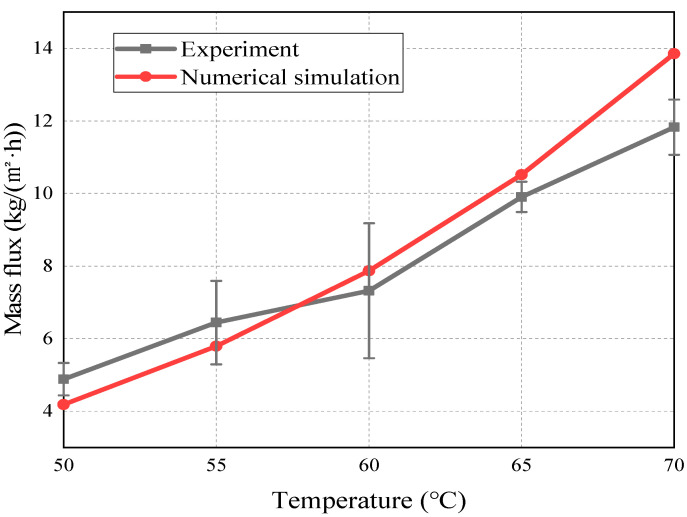
Experimental and simulation results of mass flux at different feed temperatures.

For the purpose of verification of the accuracy of the simulation results, the experimental and simulation results of the AGMD process are cross-compared when the feed liquid temperature changes from 50 to 70 °C, with the condition of the flow of feed and coolant liquid at 0.06 m/s and the inlet temperature of coolant liquid at 7 °C. In the experimental condition, the outlet temperature of the feed ranges from 46.8 to 62.7 °C, while the outlet temperature of the coolant ranges from 9.3 to 15.2 °C. The outlet temperature of the feed is significantly higher than the outlet temperature of the coolant, indicating the presence of a driving force for mass transfer across the membrane surface throughout the AGMD process. According to the Antoine equation, the saturated vapor pressure of water increases exponentially with the increase in temperature. Therefore, there exists an exponential relationship between the mass flux and the feed liquid temperature in the AGMD process. In addition, the simulation and experimental results also show exponential changes, consistent with previous results [26,27,28,29]. In addition, it can be seen from Figure 4 that the simulated value approximately coincides with the experimental value at 50–65 °C. The best fit is observed at 60 °C, with a difference of only 0.55 kg/(m^2^·h), and the worst at 70 °C, with a difference of about 2.02 kg/(m^2^·h). The reason for this is that there is heat exchange between the membrane component and the outside world during the experiment; thus, the higher the feed temperature, the greater the heat loss, causing the simulated values to be higher than the experimental values. In general, the error between the simulated value and experimental value ranges from 5.7% to 16.2%, with an average value of 11.2%, and the simulated value is in good agreement with the experimental value. The membrane contamination that may occur in the experiment is not considered in the simulation process, and saturated vapor pressure is used in the simulation instead of vapor pressure in order to ensure a consistent mass transfer driving force on both sides of the membrane, which theoretically could lead to high simulated values of mass flux. Due to the idealized conditions used in the numerical simulation, some deviations may occur between the simulated and experimental values.

#### 5.2.2. Outlet Temperature at Different Feed Flow Rates

**Figure 5:**
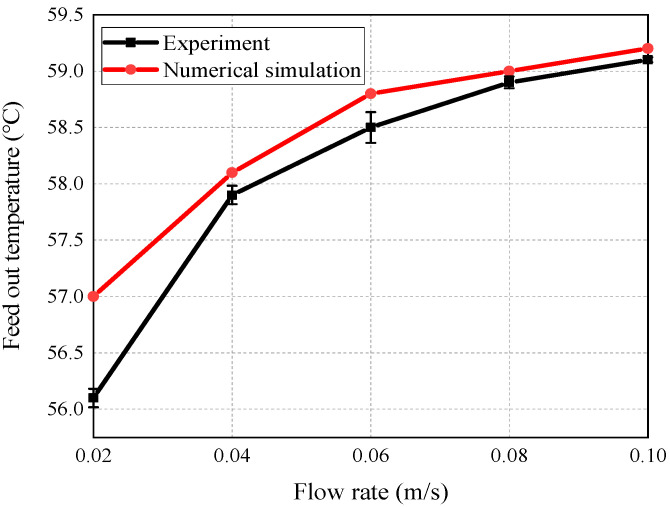
Experimental and simulation results of feed outlet temperature at different feed flow rates.

The experimental and the simulation values of the outlet temperature are cross -compared when the feed liquid flow rate changes from 0.02 to 0.10 m/s, with the conditions of the feed liquid temperature being 65 °C, the coolant liquid being 7 °C, and the initial feed concentration being 4 g/L. Figure 5 shows the outlet temperature range under different feed flow rates. It can be seen that as the flow increases, the outlet temperature of the liquid gradually rises, and then stays flat. When the flow rate is 0.02 m/s, the deviation is the largest. Enhancing the flow has been observed to decrease the boundary layer thickness and weaken the polarization, while simultaneously augmenting the mass transfer rate at the membrane surface. Therefore, the error is larger with a lower flow rate and smaller with a higher flow rate. The average error between the simulated and experimental values is 2.63%, indicating that the model can accurately simulate the process of mass and heat transfer in the AGMD system.

### 5.3. Simulation of Heat Transfer Process in AGMD

**Figure 6:**
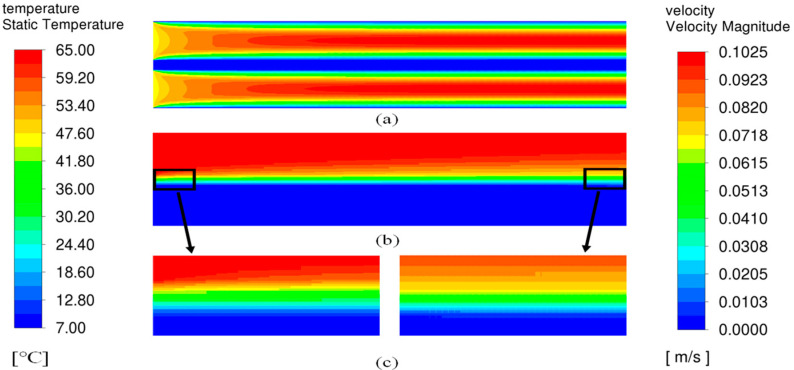
Velocity vector map and temperature cloud map of AGMD model: (**a**) velocity, (**b**) temperature, (**c**) temperature partial view.

Figure 6 shows the velocity vector map and temperature cloud map of the AGMD model. To provide a clear view, the figures have been zoomed in (5 times amplification in the y direction). In Figure 6a, the fluid velocity in the membrane module is a conventional parabola, as the fluid is affected by the walls on both the feed and the coolant channel. Due to the identical structure of the hot-side and cold-side flow channels within the membrane module, as well as with the same inlet velocity, the velocity cloud map displayed in Figure 6a exhibits a symmetrical pattern. The air in the air gap remains stationary with a velocity close to 0.00 m/s. In addition, it can be seen from Figure 6b,c that a temperature boundary layer is formed near the membranes of both the coolant side and the feed side. The main body temperature of the feed and coolant liquid remains constant. However, the membrane surface of the coolant side is obviously higher than the main body in terms of temperature. Due to the evaporation of water from the membrane surface, there is an obvious temperature difference between the membrane surface and the main body of the feed liquid on the feed liquid layer. The feed liquid layer and the temperature boundary layer of the coolant side increase gradually along the axial direction of the membrane surface and reach the maximum values at the outlet.

**Figure 7:**
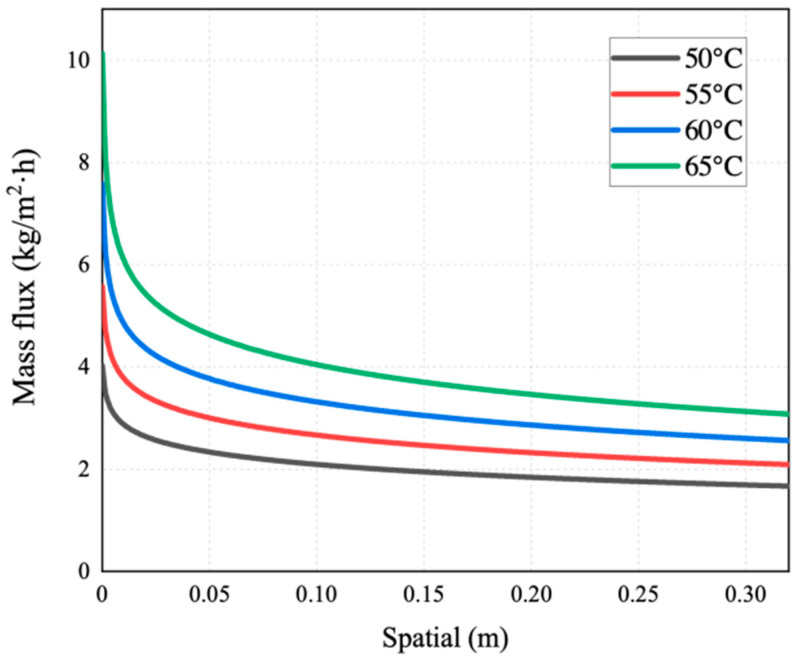
Mass flux distribution of AGMD at different feed temperatures.

**Figure 8:**
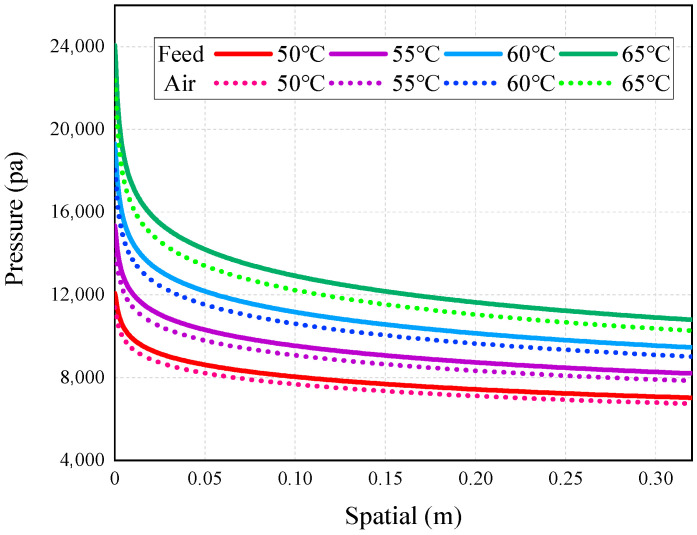
Vapor pressure distribution on both sides of the membrane.

**Figure 9:**
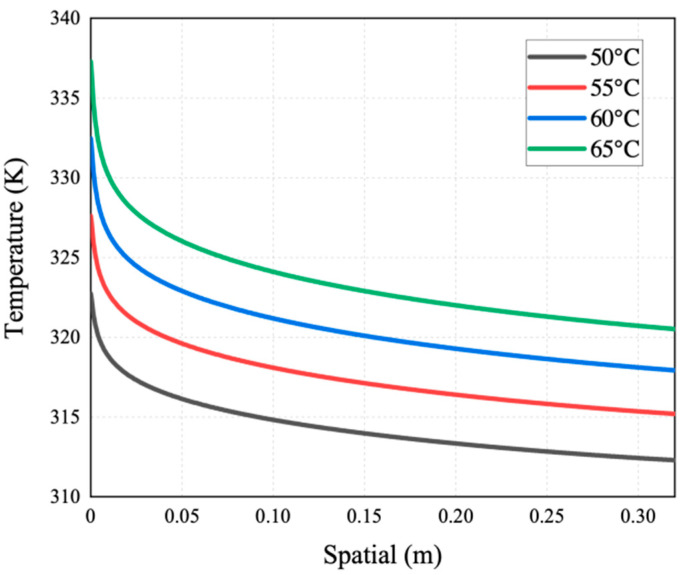
Axial distribution of membrane surface temperatures on the feed side at different feed temperatures.

**Figure 10:**
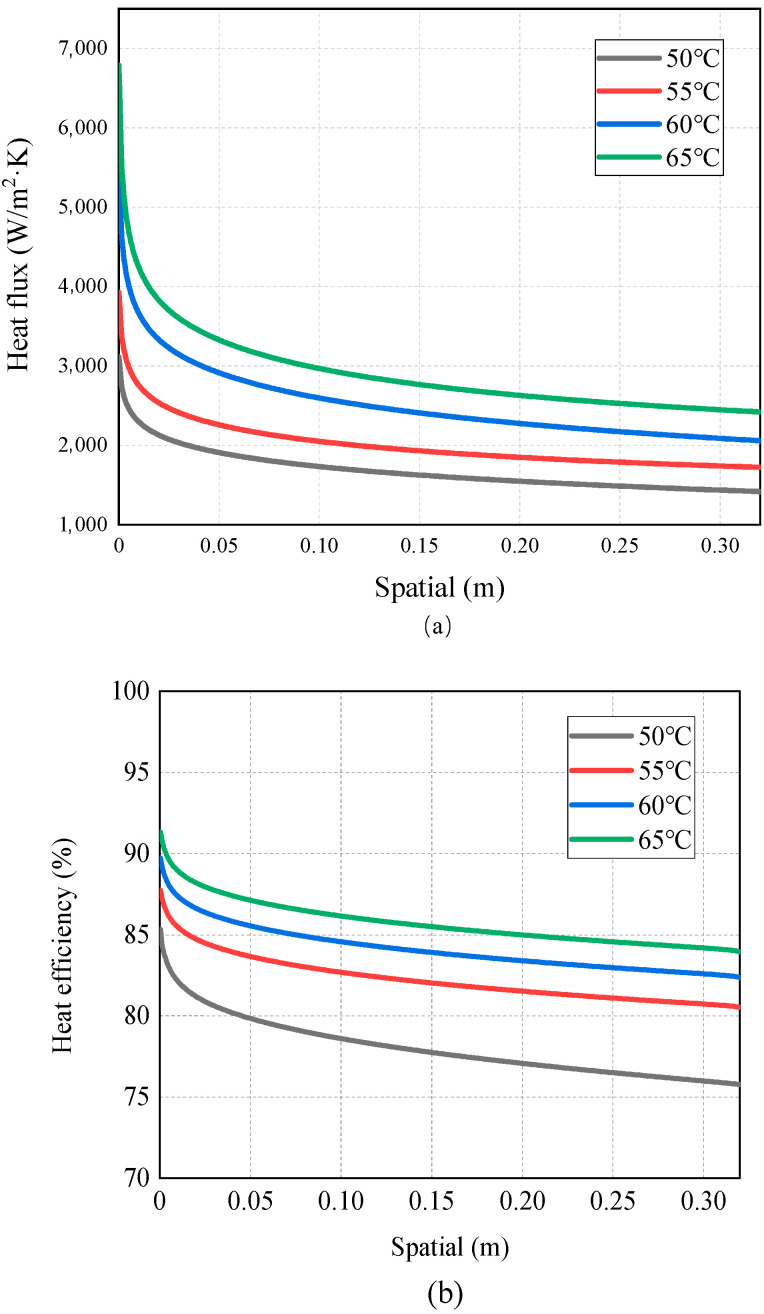
(**a**) Heat flux and (**b**) thermal efficiency distribution of AGMD at different feed temperatures.

**Figure 11:**
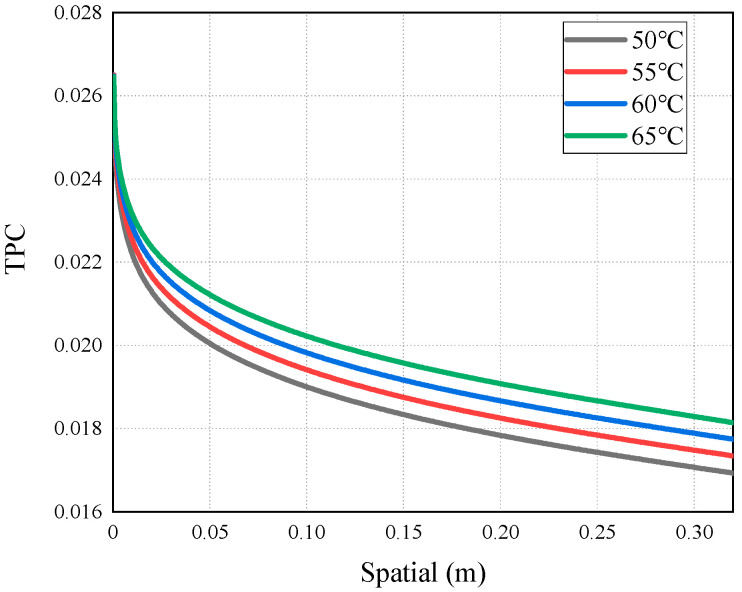
TPC distribution at different feed temperatures.

**Figure 12:**
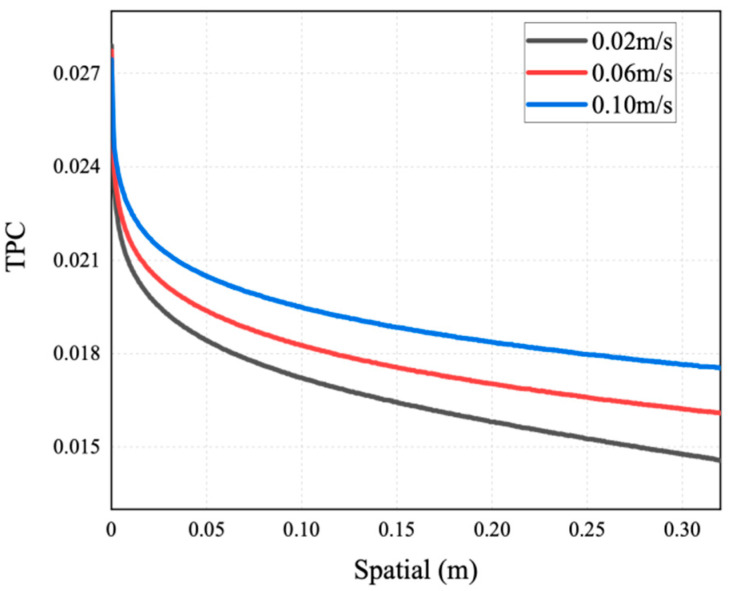
TCP distribution of AGMD at different feed velocities (under conditions of feed temperature of 65 °C and coolant temperature of 7 °C).

**Figure 13:**
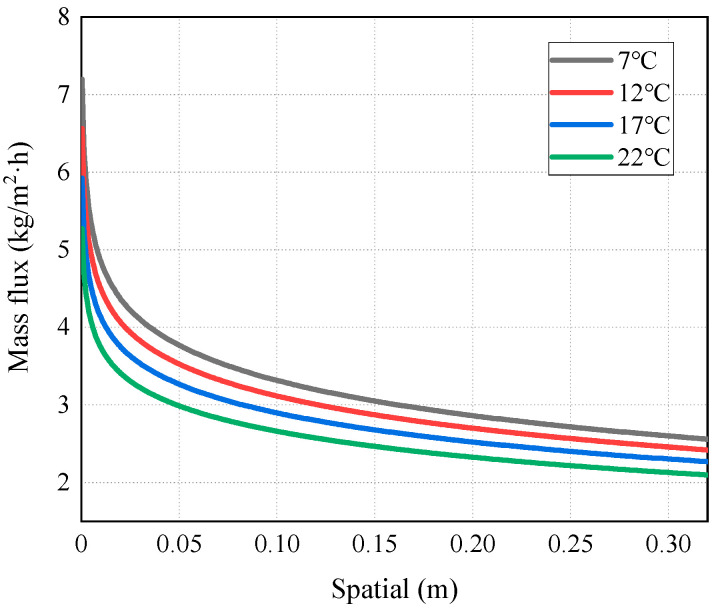
Mass flux distribution of AGMD at different coolant temperatures (under conditions of feed temperature of 65 °C and velocity of 0.06 m/s).

**Figure 14:**
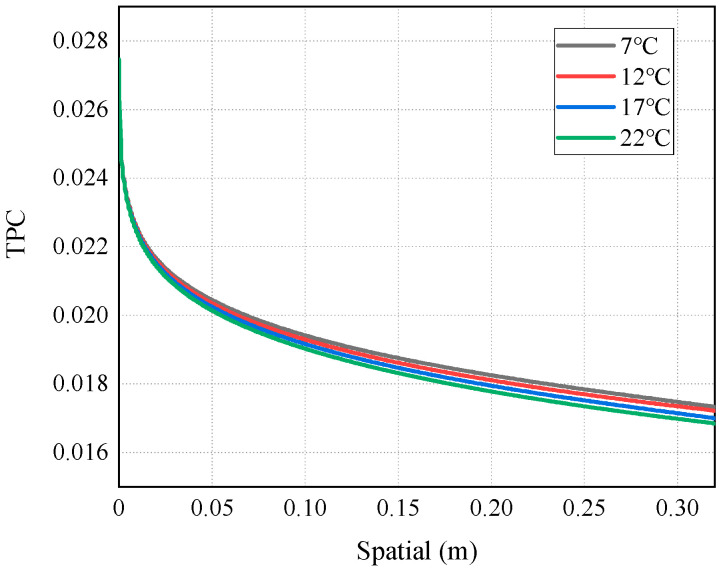
TCP distribution of AGMD at different coolant temperatures.

**Figure 15:**
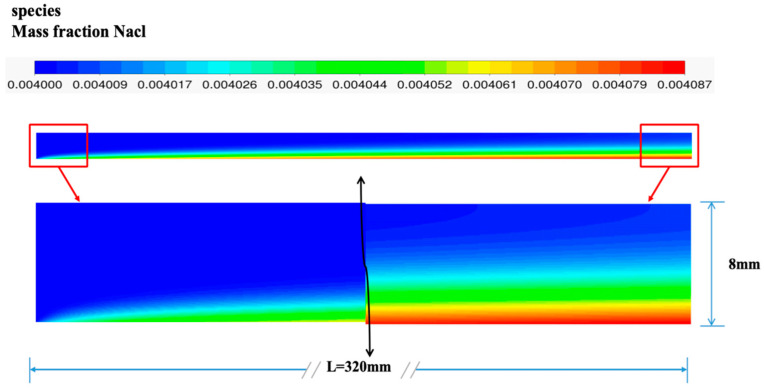
Mass fraction distribution in the feed channel.

**Figure 16:**
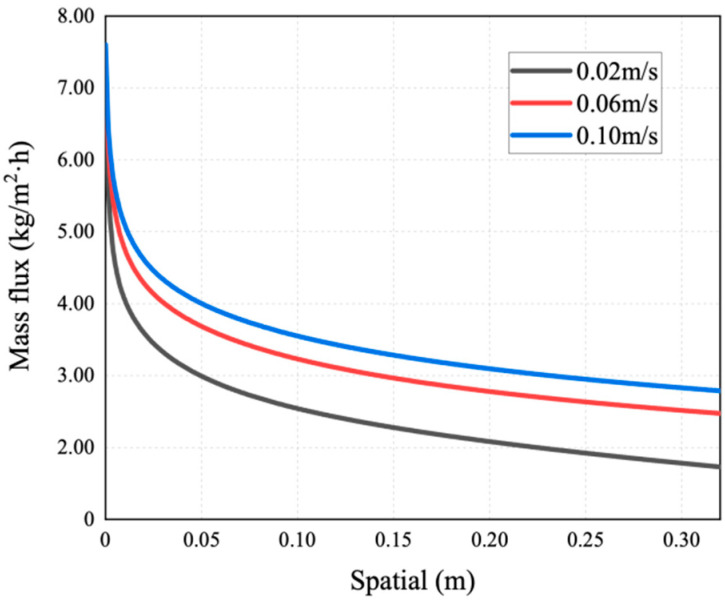
Mass flux distribution of AGMD at different feed velocities.

**Figure 17:**
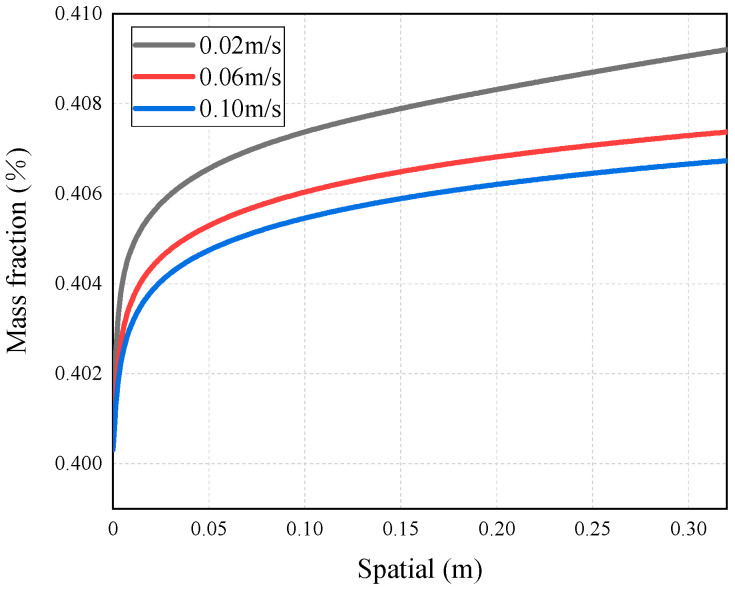
Mass fraction distribution of AGMD at different feed velocities.

**Figure 18:**
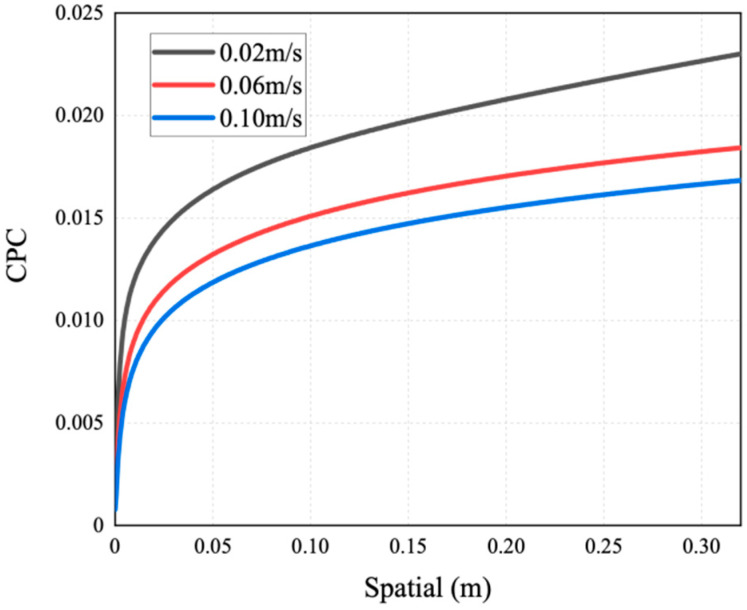
CPC distribution of AGMD at different feed velocities.

Figure 7, Figure 8, Figure 9, Figure 10 and Figure 11 show the heat transfer process simulation result when feed liquid inlet temperature ranges from 50 °C to 65 °C under the conditions of coolant temperature of 7 °C and feed velocity of 0.06 m/s. The spatial (m) in Figure 7, Figure 8, Figure 9, Figure 10, Figure 11, Figure 12, Figure 13, Figure 14, Figure 15, Figure 16, Figure 17 and Figure 18 represents the module length. It can be seen from Figure 7 that temperature has a great influence on the mass flux. The flux increases with the feed temperature [10]. The mass flux at 65 °C is increased by 81.69% compared with 50 °C. The greater the vapor pressure difference, the stronger the driving force for mass transfer. Equation (11) shows an exponential relationship between temperature and vapor pressure. Figure 8 represents the change in vapor pressure on both sides of the membrane surface. The increase in temperature leads to the increase in vapor pressure, and high-temperature feed rapidly evaporates on the membrane surface, leading to the formation of a high-concentration vapor phase, which in turn leads to the increase in mass flux. In addition, as the temperature of the feed increases, the temperature distribution within the solution becomes more even, which leads to a reduction in the temperature gradient between the feed and the membrane surface, thereby weakening the boundary layer and reducing the mass transfer resistance.

Figure 9 and Figure 10 show the distribution of membrane surface temperature and the heat flux variation at different feed temperatures. Figure 8 shows that the surface temperature of the membrane on the feed side drops rapidly near the inlet and remains unchanged after a certain distance. In the initial stage when the feed enters the module, the membrane surface temperature at the inlet is the highest. At the same time, the temperature difference is the greatest, resulting in the strongest driving force for mass transfer. As the feed flows, feed undergoes vaporization on the membrane surface, absorbing some heat. This leads to a reduction in the temperature difference and a weakening of the driving force. In addition, when the feed temperature increases, the decreasing trend in the membrane surface temperature at the inlet of the feed side gets strengthened significantly, and the membrane surface temperature at the outlet also increases. The main reason for this is that when the feed liquid-side temperature increases, the permeation flux increases, and so does the feed liquid-side membrane surface temperature and the transmembrane heat transfer flux. A temperature of 65 °C compared with 50 °C can increase the heat flux and membrane surface temperature by 118.01% and 2.83%, respectively.

The heat flux (*Q_m_*) in Figure 10a is composed of sensible heat (*Q_m_*_,*s*_) and latent heat (*Q_m_*_,*l*_). The thermal efficiency in MD typically refers to the ratio of the heat utilized (*Q_m_*_,*l*_) in the membrane process to the input heat, which can be used to evaluate the energy utilization efficiency of an MD system. Figure 10b represents the variation in thermal efficiency in the AGMD process under different feed temperature conditions. From Figure 10b, it can be observed that increasing the feed temperature increases the thermal efficiency of the AGMD process. Figure 7 and Figure 9 demonstrate that both mass flux and membrane surface temperature increase with the feed temperature, which leads to higher *Q_m,l_* and *Q_m,s_*, respectively. However, *Q_m_*_,*l*_ increases more at higher temperatures, indicating a higher energy utilization rate and higher thermal efficiency. Isam Janajreh et al. [30] modeled the effect of different operating conditions on the performance of DCMD and showed that high temperature and high flow rate increase the thermal efficiency, which is consistent with the results of this study. However, the average thermal efficiency of DCMD is only 45.1%, whereas the average thermal efficiency of AGMD in this research is significantly higher than that, reaching up to 85.7%. This shows that high thermal efficiency is the one of the advantages of AGMD.

As mentioned above, the occurrence of temperature polarization results in a decline in the AGMD process performance. TPC is an important parameter to measure temperature polarization. It can be seen from Figure 11 that with the increase in the inlet temperature of the feed liquid, the TPC value increases and the polarization phenomenon degrades. The reason for this is that increasing the feed temperature can reduce the temperature difference between the solution and the membrane surface. This brings the temperature of the membrane surface closer to the temperature of the bulk fluid, allowing more heat to be transferred to the membrane surface; thus, the formation and thickness of the temperature boundary layer are diminished, and the mass flux increases. This phenomenon is consistent with the conclusion drawn by Phattaranawik J et al. [31]. In order to further explore the influence of temperature polarization on the MD process, Figure 12 shows that the degree of TPC reduction decreases with the increase in flow rate, and the value increases with the flow rate. We analyzed the reason for this and found that when fluid is in a laminar flow state, an increase in the flow rate will reduce the thickness of the boundary layer. At the same time, the temperature difference between the two sides of the membrane increases, leading to the higher efficiency of the MD process. At present, many membrane components choose to reduce temperature polarization by adding baffles in the flow channel [32,33].

Based on the aforementioned analysis, it is evident that the mass flux is influenced by the saturated vapor pressure difference across the membrane, which, in turn, is affected by the temperature of the cooling water and its impact on the saturated vapor pressure of water vapor in the air gap on one side of the membrane. It can be seen from Figure 13 that the mass flux decreases with the increase in the coolant inlet temperature. The reason for the decrease is that the temperature of the coolant causes the vapor pressure difference on both sides of the membrane to decrease and the driving force to decrease. Compared to the feed temperature, variations in the coolant inlet temperature have a relatively minimal impact on the mass flux. The membrane flux increased by 81.69% when the feed inlet temperature increased from 50 °C to 65 °C (see Figure 7), while the mass flux only increased by 37.06% when the coolant inlet temperature decreased from 22 °C to 7 °C. By comparing the simulation data in Figure 14 with Figure 11, it can be seen that the coolant inlet temperature also has less influence on TPC than the inlet temperature. The relatively smaller impact of the coolant temperature can be attributed to the dominant role of the heat transfer coefficient in the air gap, as well as the low sensitivity of water vapor pressure at lower temperatures [21].

### 5.4. Simulation of AGMD Mass Transfer Process

MD is a process that involves simultaneous heat and mass transfer, which interact with each other. Usually, the temperature boundary layer and the concentration boundary layer would appear simultaneously. The concentration boundary layer is the difference between the main body of the feed and membrane surface concentration, which will lead to the high-concentration solute diffusing towards the lower-concentration one. The high-concentration solute on the surface of the membrane may reach the saturation state, resulting in precipitation blocking the membrane hole or mass transfer resistance, which would affect the normal progression of the MD process. Figure 15 shows the distribution of mass fraction inside the entire module when the feed temperature is set at 60 °C and the flow rate 0.06 m/s. In the MD process, only water vapor molecules can penetrate the membrane surface, whereas non-volatile components cannot. Therefore, with the ongoing reaction process, the saline concentration gradually increases along the axial direction of the membrane surface and reaches its maximum (4.072 g/L) at the outlet. The supersaturation of the main body of the feed liquid remains unchanged. The coolant side is pure water, so there is no concentration polarization.

This simulation has explored the influence of velocity on the concentration polarization phenomenon. Figure 16, Figure 17 and Figure 18 show the mass transfer process simulation result when feed velocity ranges from 0.02 m/s to 0.10 m/s under the conditions of feed temperature of 60 °C and coolant temperature of 7 °C. As can be seen from Figure 16, increasing the feed flow rate significantly increases the mass flux. With an increase in flow rate, the temperature at the membrane surface approaches the bulk temperature, resulting in an increase in the driving force and higher mass flux [21]. The flux at the velocity of 0.10 m/s is increased by 60.6% compared with 0.02 m/s. For MD, increasing the feed flow rate not only increases the mass flux, but also increases the possibility of membrane wetting. As shown in Figure 17, the saline concentration on the membrane surface decreases with a higher feed velocity, because the latter enhances the turbulence degree of the feed, and the concentration of non-volatile solutes at the membrane surface tends to approach the bulk concentration, thus enhancing the mass transfer of NaCl to the main body of the feed.

When the feed velocity is constant, the saline concentration increases rapidly due to the decline in velocity at the wall of the inlet area. When the feed liquid is fully developed, the rate of such decline slows down, causing the increase in concentration to become gentle. As can be seen in Figure 16 and Figure 18, the smaller the CPC at the inlet, the weaker the concentration polarization and the higher the mass flux. The reason behind this phenomenon is that with a smaller CPC value, the thickness of the concentration boundary layer is reduced, resulting in lower mass transfer resistance and higher mass flux.

In addition, it can be seen from Figure 17 that there is obvious concentration polarization at the feed liquid side, at a velocity of 0.02 m/s. In order to further determine the concentration polarization situation, Figure 18 shows the variation in CPC in the AGMD process with different feed velocities. From Figure 18, it can be seen that the CPC gradually decreases with the increase in flow velocity [34]. The reason for this is that when the liquid is in the laminar flow state, a higher flow velocity will reduce the concentration boundary layer, increase the mass transfer coefficient, and reduce the concentration difference between the membrane surface and the main body. Chen et al. [35] also reached similar conclusions.

## 6. Response Surface Methodology Analysis

### 6.1. Response Surface Methodology

Response surface methodology (RSM) aims to obtain a multivariate quadratic regression equation by fitting experimental results through a well-designed approach. The regression equation is solved to determine the optimal result and the corresponding operating conditions [36]. Dehban et al. [37] used RSM to design experiments and optimize the vapor-induced phase separation time and polymer concentration in a mixed solution. Khayet et al. [38] employed RSM to study the pervaporation process of acetonitrile–water mixtures and achieved the optimization of the permeation flux and the concentration of organic compounds in the permeate. In this study, the effects of feed temperature, coolant inlet temperature, and feed velocity on the performance of AGMD were investigated using RSM.

### 6.2. The Influence of the Interaction of Various Factors on Mass Flux

In order to investigate the impact of factors on the mass flux of AGMD and the interaction between factors, a response surface plot and a contour plot are drawn by using the fitted multivariate quadratic regression equation. The slope of the response surface and the density of the contour lines represent the effect of the interaction between factors on the response value. The significance of the interaction between the two factors on the response value increases as the slope becomes steeper or the contour lines become denser.

**Figure 19:**
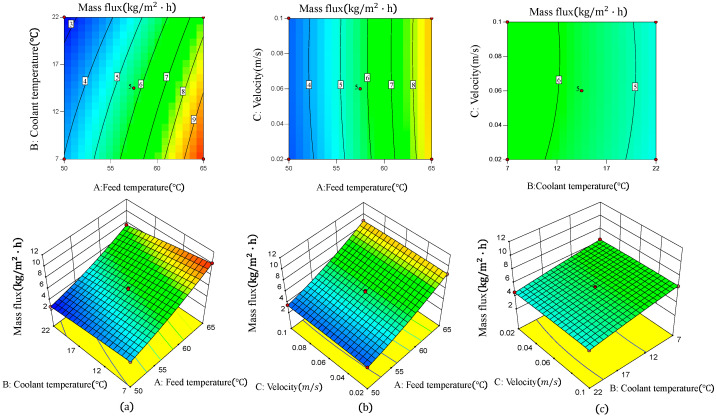
Response surface plot and contour plot of mass flux under different conditions. (**a**) Feed temperature and coolant temperature, (**b**) feed temperature and feed velocity, (**c**) coolant temperature and feed velocity.

Compared to Figure 19b,c, the response surface plot in Figure 19a has the largest surface slope, and the contours in the contour plot are also the densest. These results indicate that there is a significant interaction between feed temperature and coolant temperature. This phenomenon can be attributed to the exponential correlation between temperature and vapor pressure. The greater the temperature difference, the stronger the driving force for mass transfer [39]. Comparing Figure 19b with Figure 19a, it can be observed that although increasing the feed solution flow rate can also enhance the mass flux, its effect is not as significant as temperature [40]. The response surface plot in Figure 19c has the smallest surface slope and the sparsest contour lines in the contour plot, indicating that there is no apparent interaction between coolant temperature and feed velocity over the study range. Comparing Figure 19c with Figure 19a, it can be shown that for the same temperature difference between the feed and the permeate, the effect of the permeate temperature on the mass flux is more than 4-fold smaller than the effect of the feed temperature [21]. Due to the fact that the mass transfer performance of the AGMD process primarily depends on the vapor pressure difference across the membrane, and the presence of an air gap acts as an insulating layer, reducing the temperature of the permeate has a limited impact on the vapor pressure on the air side of the membrane. As a result, the increase in mass flux is not significant.

The variance analysis results of RSM showed that the F-values of feed temperature, coolant temperature and feed velocity are 3747.12, 437.29 and 2.32, respectively. The larger F-value represents the more significant effect of the factors. Therefore, it can be concluded that the feed temperature has the most significant effect on the mass flux, followed by the coolant temperature.

## 7. Conclusions

In this study, a two-dimensional CFD model was used to simulate the heat and mass transfer process of the flat-plate AGMD module, and the simulated values were in good agreement with the experimental values. The simulation results showed that a temperature boundary layer and a concentration boundary layer were formed near the membrane surface during the distillation process and boundary layer thickness reached the maximum values at the outlet of the AGMD module. The polarization phenomenon caused by the boundary layer reduces the driving force for mass transfer in the AGMD process, thus affecting the mass flux. Increasing the feed temperature and feed velocity can reduce the polarization effect, which corresponds to a higher TPC and lower CPC, and thus higher mass flux and thermal efficiency. The response surface methodology analysis showed that feed temperature is the most significant factor affecting the mass flux of AGMD, followed by coolant temperature and feed flow rate. There is a significant interaction between feed temperature and coolant temperature. It can be seen that increasing the feed temperature is the most effective way to improve the performance of AGMD, but it also increases the energy consumption. In the future, high flux membrane materials with corrugated surfaces and altered flow regimes within the membrane module can be used to reduce the impact of the polarization phenomenon to improve AGMD’s performance.

## Figures and Tables

**Table 1:** Membrane characteristics.

Membrane	Properties
Material	PTFE-PVDF (Polytetrafluoroethylene-polyvinylidene difluoride)
Pore	2.2 μm
Zigzag factor	1.556 (calculated by Equation (15))
Porosity	0.85
Total thickness	160 μm

**Table 2:** Boundary conditions.

	Feed		Air Gap		Coolant	
	Thermal	Velocity	Thermal	Velocity	Thermal	Velocity
Inlet	T_h_ = 50 °C, 55 °C, 60 °C, 65 °C, 70 °C	v = 0.068 m/s	Normal		T_p_ = 7 °C	v = 0.068 m/s
Outlet	P = 0, dv/dx = 0		P= 0, dv/dx = 0		P = 0, dv/dx = 0	
Top wall	Heat flux = 0	Stationary	UDF imported	Stationary	Coupled	Stationary
Lower wall	UDF imported	Stationary	Coupled	Stationary	Heat flux = 0	Stationary

**Table 3:** Physical parameters of the membrane, brine, aluminum plate, air, and water.

Material	Specific Heat	Conductivity	Density
	(J/kg·K)	(W/m·K)	(kg/m^3^)
Membrane	960	0.25	2100
Brine ^a^	4153	0.64	1017
Cold plate (aluminum)	871	202.4	2719
Air	1006.43	0.0242	1.225
Water	4182	0.6	998.2

^a^ At 4% salinity and 333 K.

**Table 4:** Mesh sensitivity.

Cell Number	Mass Flux	Error (%)	Feed Out Temperature	Error (%)
43,016	7.63	3.1	55.01	0.43
93,049	7.73	1.7	55.13	0.21
185,107	7.87	-	55.25	-
987,315	7.90	0.38	55.27	0.03

## Data Availability

The data presented in this study are available on request from the corresponding author.

## Supplementary Materials

The following supporting information can be downloaded at: https://www.mdpi.com/article/10.3390/membranes14080162/s1, the Supplementary Materials contain the UDF code.

## References

[1] Ghaffour N., Li J.D., Gray S., Francis L., Maab H., Amy G.L. (2013). Modeling of air-gap membrane distillation process: A theoretical and experimental study. J. Membr. Sci..

[2] Summers E.K., John H.L.V. (2013). Experimental study of thermal performance in air gap membrane distillation systems, including the direct solar heating of membranes. Desalination.

[3] Kumar R., Ahmed M., Bhadrachari G., Al-Missri A., Thomas J.P. (2020). The effect of chemistry of nanoparticle modifier groups on the PVDF membranes for membrane distillation. Chem. Eng. Res. Des..

[4] Zare S., Kargari A. (2022). CFD simulation and optimization of an energy-efficient direct contact membrane distillation (DCMD) desalination system. Chem. Eng. Res. Des..

[5] Ding Z., Ma R., Fane A.G. (2003). A new model for mass transfer in direct contact membrane distillation. Desalination.

[6] Phattaranawik J., Jiraratananon R. (2001). Direct contact membrane distillation: Effect of mass transfer on heat transfer. J. Membr. Sci..

[7] Khayet M. (2011). Membranes and theoretical modeling of membrane distillation: A review. Adv. Colloid Interface Sci..

[8] Cheng L.H., Wu P.C., Chen J. (2009). Numerical Simulation and Optimal Design of AGMD-Based Hollow Fiber Modules for Desalination. Ind. Eng. Chem. Res..

[9] Gopi G., Vasanthkumar M., Arthanareeswaran G., Ismail A.F., Thuyavan Y.L., Goh P.S., Matsuura T. (2023). Performance, energy and economic investigation of airgap membrane distillation system: An experimental and numerical investigation. Desalination.

[10] Martin K.A. (2011). Membrane distillation and applications for water purification in thermal cogeneration plants. Sep. Purif. Technol..

[11] Makanjuola O., Lalia B., Janajreh I., Hashaikeh R. (2022). Numerical and experimental investigation of thermoelectric materials in direct contact membrane distillation. Energy Convers. Manag..

[12] Janajreh I., Kadi K.E., Hashaikeh R., Ahmed R. (2017). Numerical investigation of air gap membrane distillation (AGMD): Seeking optimal performance. Desalination.

[13] Tan Y.Z., Ang E.H., Chew J.W. (2018). Metallic Spacers to Enhance Membrane Distillation. J. Membr. Sci..

[14] Ali K., Arafat H.A., Hassan Ali M.I. (2023). Detailed numerical analysis of air gap membrane distillation performance using different membrane materials and porosity. Desalination.

[15] Karbasi E., Karimi-Sabet J., Mohammadi-Rovshandeh J., Ali Moosavian M., Ahadi H., Amini Y. (2017). Experimental and numerical study of air-gap membrane distillation (AGMD): Novel AGMD module for Oxygen-18 stable isotope enrichment. Chem. Eng. J..

[16] Darman M., Niknafs N., Jalali A. (2023). Effect of wavy corrugations on the performance enhancement of direct contact membrane distillation modules: A numerical study. Chem. Eng. Process..

[17] Cipollina A., Miceli A.D., Koschikowski J., Micale G., Rizzuti L. (2009). CFD simulation of a membrane distillation module channel. Desalination Water Treat..

[18] Choi S.H., Tasselli F., Jansen J.C., Barbieri G., Drioli E. (2010). Effect of the preparation conditions on the formation of asymmetric poly(vinylidene fluoride) hollow fibre membranes with a dense skin. Eur. Polym. J..

[19] Imdakm A.O., Matsuura T. (2004). A Monte Carlo simulation model for membrane distillation processes: Direct contact (MD). J. Membr. Sci..

[20] Zhang J., Li J.D., Duke M., Xie Z., Gray S. (2010). Performance of asymmetric hollow fibre membranes in membrane distillation under various configurations and vacuum enhancement. J. Membr. Sci..

[21] Matsuura T., Khayet M. (2011). Membrane Distillation Principles and Applications.

[22] Ibrahim S.S., Alsalhy Q.F. (2013). Modeling and simulation for direct contact membrane distillation in hollow fiber modules. AIChE J..

[23] De Záarate J.M.O., Velázquez A., Peña L., Mengual J.I. (1993). Influence of Temperature Polarization on Separation by Membrane Distillation. Sep. Sci. Technol..

[24] Lawson K.W., Lloyd D.R. (2015). Membrane distillation. II. Direct contact MD. J. Membr. Sci..

[25] Versteeg H.K., Malalasekera W. (1995). An Introduction to Computational Fluid Dynamics.

[26] Alkhudhiri A., Darwish N., Hilal N. (2012). Membrane distillation: A comprehensive review. Desalination.

[27] El-Bourawi M.S., Ding Z., Ma R., Khayet M. (2006). A framework for better understanding membrane distillation separation process. J. Membr. Sci..

[28] Lawson K.W., Lloyd D.R. (1997). Membrane distillation: Review. J. Membr. Sci..

[29] Yu H., Yang X., Wang R., Fane A.G. (2011). Numerical simulation of heat and mass transfer in direct membrane distillation in a hollow fiber module with laminar flow. J. Membr. Sci..

[30] Janajreh I., Hussain M.N., Hashaikeh R., Rizwan A. (2018). Thermal efficiency enhancement of the direct contact membrane distillation: Conductive layer integration and geometrical undulation. Appl. Energy.

[31] Phattaranawik J., Jiraratananon R., Fane A.G. (2003). Heat transport and membrane distillation coefficients in direct contact membrane distillation. J. Membr. Sci..

[32] Anqi A.E., Usta M., Krysko R., Lee J.-G., Ghaffour N., Oztekin A. (2020). Numerical study of desalination by vacuum membrane distillation—Transient three-dimensional analysis. J. Membr. Sci..

[33] Omar A., Li Q., Nashed A., Guan J., Dai P., Taylor R.A. (2021). Experimental and numerical investigation of a new hollow fiber-based multi-effect vacuum membrane distillation design. Desalination.

[34] Ullah S.Z., Muhammad A., Sohaib Q., Younas M., Yuan Z.-H., Rezakazemi M. (2023). CFD simulation of osmotic membrane distillation using hollow fiber membrane contactor: Operating conditions and concentration polarization effects. Chem. Eng. Res. Des..

[35] Chen T.C., Ho C.D., Yeh H.M. (2009). Theoretical modeling and experimental analysis of direct contact membrane distillation. J. Membr. Sci..

[36] Fatriasari W., Ulwan W., Aminingsih T., Sari F.P., Fitria, Suryanegara L., Iswanto A.H., Ghozali M., Kholida L.N., Hussin M.H. (2021). Optimization of maleic acid pretreatment of oil palm empty fruit bunches (OPEFB) using response surface methodology to produce reducing sugars. Ind. Crops Prod..

[37] Dehban A., Kargari A., Ashtiani F.Z. (2020). Preparation and optimization of antifouling PPSU/PES/SiO_2_ nanocomposite ultrafiltration membranes by VIPS-NIPS technique. J. Ind. Eng. Chem..

[38] Khayet M., Cojocaru C., Zakrzewska-Trznadel G. (2008). Response surface modelling and optimization in pervaporation. J. Membr. Sci..

[39] Abdelkader S., Boubakri A., Geissen S.U., Bousselmi L. (2018). Direct contact membrane distillation applied to saline wastewater: Parameter optimization. Water Sci. Technol..

[40] Ali E., Orfi J., Najib A., Hamdaoui O. (2020). Understanding the dynamic behavior and the effect of feeding policies of a direct contact membrane distillation for water desalination. Chem. Eng. Commun..

