# Supplementary material for: Research on the Performance and Computational Fluid Dynamics Numerical Simulation of Plate Air Gap Membrane Distillation Module
Source: Membranes (Basel). 2024 Jul 24;14(8):162. doi: 10.3390/membranes14080162 (PMC11356412; doi:10.3390/membranes14080162)
Supplement: Supplementary file 1 [file membranes-14-00162-s001.zip › membranes-3076987-supplementary.pdf]

The UDF code:

```
#include "udf.h"
#define r 2.2e-7
#define tor 1.556
#define porosity 0.85
#define height 1e-4
#define kc 0.0546
#define b 1.6e-4
DEFINE_INIT(init_udm, domain)
{	Thread *thread_membrane = Lookup_Thread(domain,5);
	Thread *thread_membraneshadow = Lookup_Thread(domain,19);
	Thread *thread_feed = Lookup_Thread(domain,7);
	Thread *thread_air = Lookup_Thread(domain,8);
	face_t face;
	cell_t cell;
	begin_c_loop(cell, thread_feed)
{
	C_UDMI(cell, thread_feed, 0) = -1;
  }
	end_c_loop(cell, thread_feed)
	begin_c_loop(cell, thread_air)
{
	C_UDMI(cell, thread_air, 0) = -1;
}
	end_c_loop(cell, thread_air)
	begin_f_loop(face, thread_membrane)
{
	C_UDMI(F_C0(face, thread_membrane), THREAD_T0(thread_membrane), 0) = F_C0(face,
thread_membraneshadow);
      C_UDMI(F_C0(face, thread_membraneshadow), THREAD_T0(thread_membraneshadow), 0)
= F_C0(face, thread_membrane);
}
	end_f_loop(face, thread_membrane)
}
DEFINE_SOURCE(feed_water_source, cell_feed, thread_feed, dS, eqn)
{
	real source, temp0, temp1, mean_t, xm, xa, rw, p0, pa, pc, k1, k2, km, jv, TPC;
	Domain *domain = Get_Domain(1);
	Thread *thread_air = Lookup_Thread(domain, 8);
	cell_t cell_air;
		if (C_UDMI(cell_feed, thread_feed, 0) == -1)
		{
			source = 0;
			C_UDMI(cell_feed, thread_feed, 1) = source;
```

```
        }
        else
        {
            cell_air = C_UDMI(cell_feed, thread_feed, 0);
            temp0 = C_T(cell_air, thread_air);
            temp1 = C_T(cell_feed, thread_feed);
            mean_t = (temp0 + temp1) / 2;
            xm = C_YI(cell_feed, thread_feed, 1);
            xa = 18 * xm / (58.5 - 40.5 * xm);
            rw = 1 - 0.5 * xa - 10 * xa * xa;
            p0 = exp(23.1964 - 3816.44 / (temp1 - 46.13) );
            pc = exp(23.1964 - 3816.44 / (temp0 - 46.13) );
            k1 = 1.064 * r * porosity / tor / b * pow(0.018 / 8.314 / mean_t, 0.5);
            k2 = porosity / tor / b * (0.018 / 8.315 / mean_t) * 1.895e-5 * pow(mean_t, 2.072) /
1.01e5;
            km = 1 / (1 / k1 + 1 / k2);
            jv = - km * (p0 - pc);
            source = - km * (p0 - pc) / height;
            TPC = (temp1 - temp0) / (343 - 278);
            C_UDMI(cell_feed, thread_feed, 1) = source;
            C_UDMI(cell_feed, thread_feed, 2) = temp0;
            C_UDMI(cell_feed, thread_feed, 3) = temp1;
            C_UDMI(cell_feed, thread_feed, 4) = mean_t;
            C_UDMI(cell_feed, thread_feed, 5) = xm;
            C_UDMI(cell_feed, thread_feed, 6) = xa;
            C_UDMI(cell_feed, thread_feed, 7) = rw;
            C_UDMI(cell_feed, thread_feed, 8) = p0;
            C_UDMI(cell_feed, thread_feed, 9) = pc;
            C_UDMI(cell_feed, thread_feed, 10) = k1;
            C_UDMI(cell_feed, thread_feed, 11) = k2;
            C_UDMI(cell_feed, thread_feed, 12) = km;
            C_UDMI(cell_feed, thread_feed, 13) = jv;
            C_UDMI(cell_feed, thread_feed, 17) = TPC;
        }
    dS[eqn] = 0.0;
    return source;
}
DEFINE_SOURCE(memshadow_heat_flux, cell_feed, thread_feed, dS, eqn)
{
    real source, temp0, temp1, mean_t, xm, xa, rw, p0, pa, pc, k1, k2, km, jv, qr, Qc;
    Domain *domain = Get_Domain(1);
    Thread *thread_air = Lookup_Thread(domain, 8);
        cell_t cell_air;
        if (C_UDMI(cell_feed, thread_feed, 0) == -1)
```

```
            {
                source = 0;
                C_UDMI(cell_feed, thread_feed, 14) = source;
            }
            else
            {
                cell_air = C_UDMI(cell_feed, thread_feed, 0);
                temp0 = C_T(cell_air, thread_air);
                temp1 = C_T(cell_feed, thread_feed);
                mean_t = (temp0 + temp1) / 2;
                xm = C_YI(cell_feed, thread_feed, 1);
                xa = 18 * xm / (58.5 - 40.5 * xm);
                rw = 1 - 0.5 * xa - 10 * xa * xa;
                p0 = exp(23.1964 - 3816.44 / (temp1 - 46.13) );
                pc = exp(23.1964 - 3816.44 / (temp0 - 46.13) );
                k1 = 1.064 * r * porosity / tor / b * pow(0.018 / 8.314 / mean_t, 0.5);
                k2 = porosity / tor / b * (0.018 / 8.315 / mean_t) * 1.895e-5 * pow(mean_t, 2.072) /
1.01e5;
                km = 1 / (1 / k1 + 1 / k2);
                jv = km * (p0 - pc);
                qr = (-0.001351 * temp1 * temp1 - 1.4461 * temp1 + 2986.5) * 1e+3;
                Qc = (kc / b) * (temp1 - temp0);
                source = - (jv * qr + Qc) / height;
                C_UDMI(cell_feed, thread_feed, 14) = source;
                C_UDMI(cell_feed, thread_feed, 15) = qr;
                C_UDMI(cell_feed, thread_feed, 16) = Qc;
            }
        dS[eqn] = 0.0;
        return
```
